# Medication adherence in Elderly during COVID-19 pandemic: what role can the emergency department play?

**DOI:** 10.11604/pamj.2021.38.220.26555

**Published:** 2021-02-25

**Authors:** Nourelhouda Nouira, Wided Bahria, Dhekra Hamdi, Amira Lahouegue, Wiem Demni, Maamoun Ben Cheikh

**Affiliations:** 1 Mongi Slim Academic Hospital, Emergency Department, Faculty of Medicine of Tunis, University of Tunis El Manar, Tunis, Tunisia

**Keywords:** Elderly, medication, adherence, COVID-19, emergency

## Abstract

**Introduction:**

elderly frequently present a poly-pathology recurring polypharmacy. Therefore, strict medication adherence is essential to avoid poor health outcomes especially during health crises like the current COVID-19 pandemic. The aims of our study were to identify the predictors of medication non-adherence in elderly and to expose the role of the emergency department (ED) to improve the therapeutic adherence during COVID-19 pandemic.

**Methods:**

it was a two steps study. Primary, an observational, prospective survey over one month, including 100 elderly patients consulting to the emergency department. Medication adherence was assessed by Morisky's 4-questions scale; predictors of non-adherence have been identified. Secondary, a report of elderly medication management by the emergency physicians during the COVID-19 pandemic confinement.

**Results:**

first step: 100 patients, mean age of 73±8 years. The average number of drugs was 4±2. Medication non-adherence was reported in 39%, predictors of therapeutic non-adherence were: polypharmacy (OR=2.41; CI95% [1.60;3.61]), rural origin (OR=6.72; CI95% [1.47;30.63]) and metabolic diseases history (OR=5.24; CI95% [1.48;18.53]). In the second step, 816 elder lies were enrolled, mean age: 73±7 years. The therapeutic attitude in the emergency department was to prescribe the same treatment (60%) to adjust the doses of the drugs prescribed (14%) to stop one or more drugs (13%) or to indicate new treatments (13%). Thirty-five percent of patients were admitted for short-term hospitalization.

**Conclusion:**

medication non-adherence is common in elderly, due to several factors. During the COVID-19 pandemic, the emergency services in Tunisia played an important role in the follow-up and therapeutic continuity of these elderly patients.

## Introduction

Aging is considered as a real worldwide public health problem. It is the consequence of the increase in life expectancy and the decline in the fertility rate [1]. In 2015, the world's population aged 60 years and older was about 900 million, according to the last report of the World Health Organization (WHO) by 2050 elderly world population is expected to total 2 billion [1]. Elderly frequently present a poly-pathology source of polypharmacy, a study had shown that 75% of elderly aged 70 to 74 years old take drugs, this proportion rises to 88% in patients over 85 years [2]. The number of prescription drugs increases with age. Indeed, prescriptions contain an average of 3 to 4 drugs for patients aged 65 to 70 years, compared to 3 to 6 drugs after 70 years; for these age range, 30% have five or more medications [3,4]. The polypharmacy with multiple prescribing physicians can decrease the adherence to the treatments [5], in the literature, non-adherence in the elderly varies between 20% and 40% and can reach 60% in patients with severe medical history especially cardiovascular diseases [6]. In 2017, a Tunisian study had shown that elderly medication non-adherence was observed in 34% of patients followed in the internal medicine consultation [7]. Consequently, non-adherence to the treatments can result in an avoidable intensification of treatments and increase iatrogenic risk in elderly. The current COVID-19 pandemic has exacerbated the problems that were already causing people to not take their drugs [8]. With the continued lockdowns, the social distancing and the economic hardships, non-adherence is likely to get worse especially in elderly. In the absence of specialized services in geriatrics and of Mobile Geriatric Teams in Tunisia, the aged patients frequently visit emergency departments (ED) for urgent and less urgent care where the emergency physician could play a major role in adjusting the treatment of these patients.

The aim of our study was: to identify the predictors of medication non-adherence in a sample of aged patients and to assess the role of the emergency department to improve the medication adherence in elderly during the COVID-19 pandemic.

## Methods

### Assessment of medication adherence in elderly sample consulting the emergency department before the COVID-19 first-wave in Tunisia

**Setting:** the first stage of this study was a prospective observational, monocentric survey with descriptive and analytic approaches. Over one month before the COVID-19 first-wave in Tunisia, from November 20^th^, 2019 to December 20^th^, 2019 at the ED of Mongi Slim Academic Hospital, which is the sole University Hospital Center serving a population of approximately 200,000; its emergency department had an annual volume of 70,000 visits.

**Participants:** one hundred patients were included. The study inclusion criteria were the age of 65 years or older and a history of drug use before the consultation in the ED, whatever the reason of consultation. Elderly patients with neurological impairment, difficult history taking and who required an immediate support in the emergency room were not included.

**Procedure:** medical history with the data related to the treatment were collected: the number of drugs on the daily prescription of the elderly, the knowledge of the different medications, the pharmaceutical classes, the nature of prescriptions (long-term, punctual, self-medication) and the rhythm of consultations. In this study the validated French version of the four-questions “Morisky Medication Adherence Scale” (MMAS) [9,10] was used to assess the medication adherence of the elderly. The MMAS consists of four items with as coring scheme of “Yes” = 0 and “No” = 1. The four questions were: “Do you ever forget to take your (name of health condition) medicine?”, “Do you ever have problems remembering to take your (name of health condition) medication?”, “When you feel better, do you sometimes stop taking your (name of health condition) medicine?”, “Sometimes if you feel worse when you take your (name of health condition) medicine, do you stop taking it?”. The items are summed to give a range of scores from 0 to 4, the patient who had a score of zero is considered adherent to his medication, who had 1 or 2 is considered moderately adherent and patients with 3 or 4 points on this scale were considered as non-adherent. The impact of polypharmacy and of other factors on adherence to treatment was assessed.

### Medications management for elderly patients in the emergency department during the COVID-19 pandemic

**Participants:** all the elderly patients, 65 years or older, consulting the emergency department of Mongi Slim University Hospital during the COVID-19 pandemic confinement in Tunisia, from March 25^th^ to June 4^th^ 2020.

**Procedure:** demographic data, medical history, medications, reason for consultation in the emergency service and the last medical appointment were collected. All prescriptions have been reviewed, patients stopping or at risk of stopping their medication have been targeted. The possible attitudes of the emergency doctor to elderly drug treatment, according to the patient's condition, were: to keep the same treatment, to renew the same prescription that was stopped, to stop one or more treatments, to adjust the doses and to prescribe a new treatment. The decision to hospitalize the patient or not was also noted.

**Statistical analysis:** we used the Statistical Package for Social Sciences (SPSS 22.0, IBM) for analysis. The comparison of two independent series were made using the Student's Independent T-test and in case of non-validity, by the non-parametric Mann-Whitney test. The comparison of percentages was made by the Pearson Chi-square test, and in case of non-validity of this test, by Fisher's exact test. The value of p < 0.05 was considered significant. The search for risk factors was performed by calculating the Odds Ratio (OR). We conducted a multivariate analysis in logistic regression. An adjusted OR highlighting the role of the factor was calculated. A 95% confidence interval (CI) was used.

**Ethical considerations:** patients were informed about the purpose of this study before the data collection; an informed oral consent was obtained from each of the participants. The data collection was anonymous, the medical confidentiality as well as the protection of the personal data of the patients were respected.

## Results

**Assessment of medication adherence in elderly sample consulting the emergency department before the COVID-19 first-wave in Tunisia:** one hundred patients aged 65 years or older were enrolled in this survey. The average age was 73±8 years (65 to 102). Sex-ratio was 1.43. Eighty-two percent of the patients were from urban areas. The cardiovascular and metabolic diseases were the most frequent medical history.

**Drug use:** in our study, 49% of the elderly were able to recognize and to take their medication against 51% who were dependent of another person. The average number of medications prescribed to the elderly was 4±2 drugs with a median of 4 and a maximum of 9 treatments. The most prescribed medications for chronic diseases in our study were cardiovascular and antidiabetic drugs: angiotensin-converting enzyme (ACE) inhibitors (46%), oral antidiabetic agents (39%), acetylsalicylic acid (31%), diuretics (30%) insulin (29%) and beta blockers (28%). In addition to long-term prescription, sum patients were treated for acute pathologies and the drugs used were analgesics (12%), antibiotics (9%), proton pump inhibitors (5%) and non-steroid anti-inflammatory (4%). According to the MMAS, 39% of the elderly were non-adherents to their medications (Table 1). The median number of medications was significantly greater among non-adherent elderly (6 in non-adherent against 3 in adherent elderly, p=0.031).

**Table 1:** elderly medication adherence according to the morisky medication adherence scale (MMAS)

MMAS	%
0 (Adherent)	3
1 (Moderately Adherent)	56
2 (Moderately Adherent)	2
3 (Non-Adherent)	25
4 (Non-Adherent)	14
**Total**	100

Patients who had a score of 0, 1 or 2 is considered as adherent to his medication and patients with 3 or 4 points on this scale were considered as non-adherent

**Predictors of medication non-adherence in elderly with univariate and multivariate analysis:** we compared the “adherent” elderly group to the “non-adherent” group in demographic characteristics, medical history, prescription data and orientation (Table 2). The independent predictors of non-adherence to the medication in elderly were rural origin, metabolic history and polypharmacy.

**Table 2:** comparison between the characteristics of adherent elderly group and the non-adherent to the medication group (univariate analyze)

Characteristics	Population (n=100)	Non observants (n=39)	Observants (n=61)	OR	IC [95%]	p
Age (Years)	73±8	71±6	76±10	-	[-7.15; -0.57]	0.01
Female	61	23	18	2.86	[1.24; 6.58]	0.03
Origins						
Urban area	82	54	28	3.03	[1.05; 8.67]	0.03
Rural area	18	11	7			
Medical history						
Metabolic	58	20	38	-	-	0.27
Cardiovascular	75	32	43	-	-	0.19
Neurological	17	9	8	-	-	0.06
Respiratory	19	11	8	-	-	0.06
Prescription						
Long term	49	31	18	0.10	[0.04; 0.28]	0.01
Punctual	23	10	13	-	-	0.61
Self-medication	19	4	15	-	-	0.07
Total number of medications	4±2	5±2	3±1	-	[-3.17; -1.75]	0.01
Orientation						
Hospitalization	52	28	24	0.25	[0.10; 0.60]	0.02
Back home	48	11	37	-	-	-

n: number, OR: Odds ratio, CI: confidence interval

**Medications management for elderly patients in the emergency department during the COVID-19 pandemic:** during the COVID-19 confinement period 4606 patients consulted in the emergency department, among them 816 were 65 years or older enrolled in the study. The average age was 73±7 years (65 to 98). Sex-ratio (male/female) was 1.21. Eighty-four percent of the patients were from urban areas. The cardiovascular and metabolic diseases were the most frequent medical history, 41% of elderly patients have not consulted a doctor for more than 6 months. The majority consulted for dyspnea and chest pain, demographic characteristics and medical history of the sample (n=816) are summarized in Table 3.

**Table 3:** baseline characteristics of the elderly patients consulting the emergency department during the COVID-19 confinement (n = 816)

Characteristics	Value
Sex ratio	1.2
Age years mean (SD)	73±7
**Origins**	
Urban area	689 (84%)
Rural area	127 (16%)
**Comorbid diseases n (%)**	
Hypertension	374 (45%)
Diabetes	283 (34%)
Coronaropathy	104 (12)
Chronic heart failure	61 (7%)
Chronic renal failure	35 (4%)
**Last medical appointment**	
Less than 3 months	114 (14%)
Between 3 and 6 months	367 (45%)
More than 6 months	335 (41%)
**More than 3 medications**	
Yes	255 (31%)
No	561 (69%)
**Reasons for consultation**	
Dyspnea	281 (34%)
Chest pain	186 (23%)
Nausea, vomiting	69 (8%)
Abdominal pain	54 (7%)
Sore throat	29 (3%)
Other	197 (24%)

SD: standard deviation, COVID-19: coronavirus disease-19

**Emergency physician attitude and the orientation of the elderly:** in our study, the attitude of emergency physicians towards elderly patient´s medication, especially those who are at risk of stopping their treatment (female, with rural origin, with metabolic history or with polypharmacy) was to prescribe the same treatment (60%) or to adjust the doses of the drugs prescribed (14%) with a reminder of therapeutic education, for the patient if autonomous or for his accompanying, depending on the treatment. In some situations, the Emergency physician had made the decision to stop one or more drugs (13%) or to indicate new treatments (13%). Thirty-five percent of patients were admitted to the emergency room for short-term hospitalization and 7% were admitted to the other specialized services, while 58% returned home.

## Discussion

In Tunisia, the number of elderly, aged 65 years and over, represents 8.2% of the population, more than half of whom are aged 75 years and over with a significant variation in the age pyramid between 1988 and2018 [11,12]. Life expectancy in Tunisia increased from 73 years in 2000 to 75 years in 2017. Elderly patients frequently present a polypathology requiring the combination of many drugs concomitantly, which could have repercussions on the therapeutic adherence defined as “the degree to which the person´s behavior corresponds with the agreed recommendations from a health care provider” [13] and can be replaced by compliance or concordance. In the first step of our study, within a sample of one hundred elderly patients consulting in the Emergency Department, therapeutic non-adherence was noted in 39% of the patients. A meta-analysis published in 2019 had shown that the frequency of non-adherence in elderly hypertensive patients varied from 13% to 90% of the analyzed populations [9]. Many factors influence the maintenance of medication adherence: motivation, understanding the treatments goals and social behavior as well as cognition and functional status of the aged patient [5]. In elderly poor medication adherence results in therapeutic inefficiency, generating additional consultations and investigations and a significant cost with recurrent hospitalizations. In our study, non-adherent patients required more hospitalizations in the ED and in the other services in comparison with adherent patients (p<0.01). A German study showed that 23,5% of hospitalizations for decomposition of chronic heart failure were related to therapeutic adherence problems [14]. Three independents predictors of medication non-adherence were identified in our study: rural origin, metabolic history and the total number of drugs, Mejri et al identified 6 factors related to reduced compliance in univariate analyze: advanced age (p=0.013), low school level (p=0.034), cognitive impairment (p=0.022), poor knowledge of medical treatment (p=0.01) and physician nearby (p=0.005) [15].

Polypharmacy, defined as the use of multiple drugs administered to the same patient [16], most commonly seen in elderly patients, was identified in our study as an independent predictor of medication non-adherence. A French study published in 2015 found that 33% of polypharmacy treatments, for patients aged over than 75 years, were used on chronic diseases and 40% by incorporating a new medication of intercurrent diseases [17]. Polypharmacy could result in severe adverse effects whose may be increased by the reduction of elimination capacities and by the other consequences of ageing [18]. It was shown that 5 to 20% of hospitalizations of elderly were related to the iatrogenic risk and in 60% to drugs interactions [14]. A study had shown that the level of understanding of the treatment was reduced in more than half of the patients aged 80 years and over, that the liquid form is not appreciated by the elderly and that the discomfort of treatment were essentially related to the taste of drug, the difficulty of opening or deblistering the packages, to the size of the tablets and the difficulty of the identification of each drug [19]. For elderly patients any slackening in the medical monitoring can unbalance their pathologies. One of the unprecedented circumstances that could have a negative impact on elderly health and well-being is the COVID-19 pandemic. An exceptional universal health and social crisis which forced most countries to lockdown many cities and restrict movement within such clusters. In Tunisia, total containment was declared on March 20^th^, 2020 with the suspension of all scheduled activity in all hospital departments. Patients with chronic diseases, especially the elderly, did not know what to do with their management and medicines during this confinement. The WHO had recommended that countries should identify essential services that will be prioritized in their efforts to maintain continuity of service delivery and make strategic shifts to ensure that increasingly limited resources provide maximum benefit for the population [20]. Among these essential services are geriatric services and those who take care of the elderly. In the absence of geriatric services in Tunisia, the pandemic restraint has led to many patients relying on emergency departments for their medication prescription and health information. Where the emergency physicians played a crucial role in maintaining adherence to treatment in these elderly patients.

The weaknesses of our study were the small sample size (100 patients) from only one center in the first step survey. The adverse effects of the drugs are an important factor of non-adherence, especially in the elderly, and is often increased with the number of medications, our study did not evaluate the role of the drug iatrogenic risk on therapeutic adherence. In the second step, during the COVID-19 pandemic, elderly patients were not followed up after their visit to the emergency service to reassess the impact of the emergency doctor´s intervention on elderly medication adherence.

## Conclusion

The ageing is a real public health problem. Elderly often present a poly-pathology requiring strict medication adherence. Several factors can influence elderly adherence to treatments, especially the large number of drugs prescribed. A greater attention should be paid to supporting the therapeutic adherence in geriatric patients by reviewing the prescriptions, simplifying the therapeutic routines, adapting the doses and the numbers of daily doses and planning a drug intake. The worldwide challenge is to adjust the medical professional behavior and practices, according to the needs of elderly patients especially in unusual circumstances like a current COVID-19 pandemic. Given, medication non-adherence in elderly can drive up otherwise avoidable hospital admissions, burdening healthcare systems already struggling to care for coronavirus patients and putting other patients at greater risk of contracting COVID-19. During the confinement in Tunisia the emergency departments were the only escape route for these patients, and emergency doctors had played a crucial role in maintaining elderly therapeutic adherence.

### What is known about this topic

- Elderly patients, given their pathologies, the limitation of their autonomy and their psychological and social fragility are at risk of therapeutic non-adherence;
- During health crises, the usual medical follow-up is compromised with a decrease in the vigilance and availability of caregivers.

### What this study adds

- More than a third of elderly patients may be non-adherent to their treatment;
- Polypharmacy is the most important factor in medication non-adherence in elderly;
- Emergency services play an important role in maintaining treatment adherence in elderly through prescription verification and management.
